# Evaluating the efficacy of subthreshold micropulse laser combined with anti-VEGF drugs in the treatment of diabetic macular edema: a systematic review and meta-analysis

**DOI:** 10.3389/fendo.2025.1553311

**Published:** 2025-03-28

**Authors:** Yang Jiang, Wei He, Shixin Qi

**Affiliations:** Department of Ophthalmology, Tianjin Medical University Baodi Hospital, Tianjin, China

**Keywords:** diabetic macular edema, subthreshold micropulse laser, anti-VEGF drugs, combination therapy, meta-analysis

## Abstract

**Objective:**

To compare the clinical efficacy of subthreshold micropulse laser (SML) combined with anti-VEGF drugs versus anti-VEGF drugs monotherapy for diabetic macular edema (DME) through a meta-analysis.

**Methods:**

We systematically reviewed relevant literature from electronic databases and extracted key outcomes, including best corrected visual acuity (BCVA)—comprising ETDRS and LogMAR measures, central macular thickness (CMT), annual frequency of anti-VEGF injections, annual SML applications, and associated complications for both treatment groups at postoperative intervals of 3, 6, 9, and 12 months.

**Results:**

A total of 13 relevant studies were included in this review, including 405 eyes in the experimental group (SML combined with anti-VEGF drugs intravitreal injections) and 400 eyes in the control group (anti-VEGF drugs monotherapy). The results showed no significant difference in ETDRS visual acuity between the two groups at any time point (P > 0.05). However, LogMAR visual acuity significantly improved in the experimental group compared to controls at both 6 and 12 months post-treatment (P < 0.05). Subgroup analysis based on baseline CMT values indicated that patients with baseline CMT < 400 µm had significantly more reduced CMT across all observation points in the experimental group (P < 0.05). Conversely, no significant differences in CMT were found among those with baseline CMT ≥ 400 µm (P > 0.05). Additionally, annual intravitreal injection frequency of anti-VEGF drugs was significantly reduced in the experimental group compared to the controls (P < 0.05). The average numbers of SML applications per year ranged from 1.41 ± 0.37 to 3.4 ± 1.4 times (range: 1–4 times). Common postoperative complications included mild subconjunctival hemorrhage, a light ocular inflammatory reaction, and/or ocular surface discomfort.

**Conclusion:**

Compared to anti-VEGF drugs monotherapy, combining SML with anti-VEGF drugs can improve visual acuity and reduce macular edema among DME patients—especially those with central macular thickness < 400 µm. The combined approach also reduces anti-VEGF drugs intravitreal injection frequency, and repeated use of SML can alleviate economic burdens on patients.

**Systematic review registration:**

https://inplasy.com, identifier INPLASY2024120068.

## 1 Introduction

Diabetic macular edema (DME) is one of the most prevalent microvascular complications of diabetes. The incidence rates of DME and clinically significant macular edema (CSME) among individuals with diabetes (1) are 5.2% (3.1-7.9%) and 3.5% (1.9-6%), respectively, with projections indicating that the number of affected individuals worldwide will rise to 28.61 million by 2045 (2). DME can occur at any stage of diabetic retinopathy, impairing central vision and serving as a major cause of severe vision loss in working-age populations (2). In recent years, intravitreal injection of anti-vascular endothelial growth factor (Anti-VEGF) drugs has become the first-line treatment for diabetic macular edema (3). However, this therapy has a short duration of effect, high recurrence rates, and requires repeated injections, leading to significant economic burdens and poor patient compliance. Traditional management for DME involves retinal laser photocoagulation; although effective, this method can cause retinal tissue burns and visible retinal scars (4). In contrast to traditional continuous-wave lasers, subthreshold micropulse laser (SML) selectively targets cells of the retinal pigment epithelium (RPE), causing only sublethal damage to RPE, preserving photoreceptor cells and surrounding tissues. Consequently, this approach allows for the treatment of macular edema without noticeable retinal scars, minimizing adverse effects, and enabling multiple repeat treatments (5, 6). While the efficacy of anti-VEGF intravitreal injections remains exact, the majority of studies (7–9) suggest that combining anti-VEGF with SML is more effective than anti-VEGF monotherapy in improving visual acuity and reducing the number of injections required. Nevertheless, there are still sceptical voices that DME patients can’t benefit from the combination strategy (10). Moreover, the majority of existing studies are small-sample independent studies, which limits their potential to offer evidence-based medicine. Therefore, this study conducted a comprehensive systematic review and meta-analysis to compare the clinical efficacy of SML combined with anti-VEGF drugs versus anti-VEGF monotherapy in the treatment of DME.

## 2 Materials and methods

This systematic review was performed in accordance with the PRISMA protocol and was previously registered in the INPLASY database (https://inplasy.com/inplasy-2024-12-0068/) under the registration number INPLASY2024120068.

### 2.1 Literature screening

We conducted a search throughout multiple databases, including Pubmed, Embase, Cochrane, Web of Science, China Journal Full Text Database (CNKI), China Wipo Full Text Database (VIP), Wanfang Database, and China Biomedical Literature Database (CBM), without any language restrictions since the inception of these databases. At the same time, the references involved in the included literature were tracked, and grey literature was identified. The search terms and detailed search strategies are presented in the **Supplementary Data Sheet 1** search strategy.

### 2.2 Criteria for studies selection

Inclusion Criteria 1) Randomized controlled trial or retrospective study; 2) The study was conducted in patients >18 years of age with DME, defined as macular edema involving the macular central fovea, with no restriction on age, race, sex or duration of disease; 3) The intervention was anti-VEGF drugs intravitreal injection combined with subthreshold micropulsed laser (SML) treatment in the experimental group, and anti-VEGF drug monotherapy (or combined with sham laser treatment) in the control group; 4) Reported outcomes included at least one of the following: best corrected visual acuity (BCVA), central macular thickness (CMT), the number of anti-VEGF agents injections per year, the number of SML annual applications, and associated complications; 5) Papers reported at least one of the above outcomes at baseline (0) and at 3, 6, 9 and 12 months after treatment.

Exclusion Criteria 1) Patients who had received panretinal photocoagulation, SML, vitreous anti-VEGF injection, and any other intraocular surgeries other than cataract surgery in the previous 3 months were excluded; 2) The subjects were non-DME patients (e.g., macular edema due to other sources such as retinal vein occlusion) or fluorescein fundus angiography (FFA) examination suggestive of macular ischemia; 3) The interventions in the experimental group were either SML treatment alone or anti-VEGF drugs intravitreal injections combinated with non-SML treatment (e.g., combined with conventional retinal laser); 4) Animal experiments, case reports, conference papers, reviews, duplicate publications, literature from which full text was not available or from which original data could not be extracted.

### 2.3 Data extraction and quality assessment

The following data were extracted: 1) Author name, publication year; 2) General information about the subjects (age, sample size, glycated hemoglobin A1c (HbA1c), etc.); 3) Type of study, interventions, follow-up time, and outcome indicators. Continuous variables are presented as mean ± standard deviation (x ± s). All data collection and extraction in this paper were done independently by two researchers, and in a case of disagreement, the decision could be made through consultation or with the assistance of a third researcher.

A risk of bias assessment was undertaken for randomized controlled studies using the Cochrane Risk of Bias 2 (RoB 2) tool and for retrospective studies with the Cochrane Risk of Bias in Non-randomized Studies of Interventions (ROBINS-I) tool. Two researchers conducted the risk of bias evaluation separately, providing a rating to each item within each domain as either ‘low’, ‘some concerns/moderate’, or ‘high’ risk. The overall all of these factors resulted in the evaluation of overall bias. Any disagreement on ratings was discussed and resolved with a third reviewer.

### 2.4 Statistical analysis

Meta-analysis was completed using Revman 5.3 and Stata SE15 software provided by the Cochrane Collaboration. Since the included studies had inconsistent indicator measures or units, standardized mean difference (SMD) was served as the effect size, and each effect size was given its 95% confidence interval (95% Cl). Heterogeneity between studies was assessed using Cochran’s Q test and I^2^ statistic. When P > 0.1 and I^2^ < 50%, it was considered that there was no statistically significant heterogeneity between studies, and statistical analysis was performed using a fixed-effect model. When P < 0.1, I^2^ ≥ 50%, then it was considered that there was a statistical heterogeneity. A meta-regression analysis was taken to attempt to figure out the clinical reasons for the heterogeneity, and a subgroup analysis was performed when necessary. If no obvious reasons for clinical heterogeneity were found, Galbraith star plots were created, and a fixed-effect model was employed after eliminating the literature with large heterogeneity, or a random-effect model was adopted. Sensitivity analyses were performed using a one-by-one exclusion method. Finally, the Begg’s test for publication bias was performed.

## 3 Results

### 3.1 Literature search

**Figure 1:**
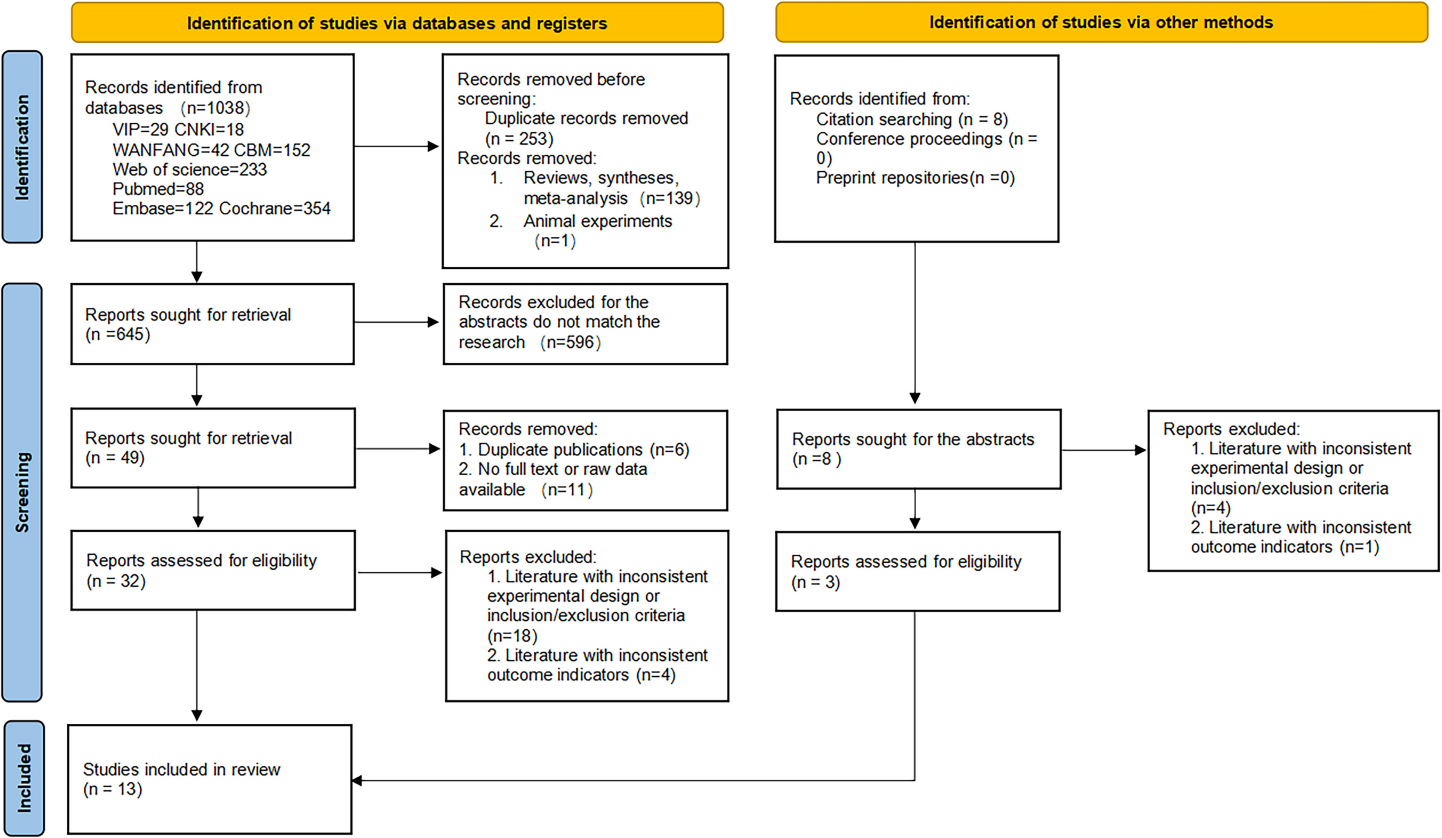
PRISMA flowchart.

A total of 1046 documents were identified by searching the database using the appropriate search strategy. 13 documents were finally included after screening, including 11 randomized clinical trials and 2 retrospective studies. There were 405 eyes in the experimental group (anti-VEGF drugs combined with SML) and 400 eyes in the control group (anti-VEGF drugs alone). There was no statistically significant difference in the baseline BCVA, baseline CMT, and baseline HbA1c levels between the two groups (all P > 0.05). However, the experimental group was older than the controls (SMD = 0.23, fixed-effect model, P = 0.002 < 0.05), which was statistically significant. The search process and results are displayed in **Figure 1**, and the basic characteristics of the included studies are provided in **Tables 1**, **2**.

**Table 1:** Basic characteristics of included literature (1).

Study	Design of Study	Number of cases(eyes)		Age (years)		Interventions		The time of follow-up (months)		Outcome
Author(year)		T	C	T	C	T	C	T	C	
Huang KQ 2022 (11)	RCT	26	26	62.31 ± 5.48	63.77 ± 5.37	SML+ranibizumab	Ranibizumab	9	9	0 and 3.6.9 mo:①②
Li WQ 2019 (15)	RCT	36	32	57.2 ± 10.1	60.6 ± 12.3	SML+conbercept	Conbercept	12	12	0 and 3.6.9.12 mo:①②③
Sun GL 2017 (12)	RCT	15	16	58.27 ± 6.85	57.69 ± 6.39	HD-SDM +ranibizumab	Ranibizumab	13.53 ± 1.72	15.19 ± 2.56	0 and 12 mo:①②③④
Wu Q 2021 (16)	RCT	36	36	56.8 ± 10.2	56.37 ± 9.5	SML+ranibizumab	Ranibizumab	9	9	0 and 3.6.9 mo:①②
Zhang Q 2021 (17)	RCT	33	31	56.0 ± 7.7	53.3 ± 9.1	SML+aflibercept	Aflibercept	12	12	0 and 3.6.12 mo: ①②③④⑤
Zheng LL 2023 (18)	RCT	30	30	57.12 ± 12.18	55.34 ± 11.86	SML+conbercept	Conbercept	6	6	0 and 3.6 mo:①②④
Zhou JX 2023 (13)	RCT	50	50	43.62± 9.94	43.87 ± 10.86	SML+conbercept	Conbercept	12	12	0 and 12 mo:①②③⑤
Abouhussein 2020 (7)	RCT	20	20	60.4 ± 4.2	59.5 ± 4.3	SML+aflibercept	Aflibercept	12	12	0 and 3.6.9.12 mo:①②③④
Khattab 2019 (9)	RCT	27	27	59.4 ± 4.3	55.7 ± 3.4	SML+aflibercept	Aflibercept	18	18	0 and 3.6.12 mo:①②④
Kanar 2019 (19)	RCT	28	28	63.42 ± 10.14	62.64 ± 9.03	SML+aflibercept	Aflibercept	12	12	0 and 3.6.12 mo:①②③⑤
Altınel 2021 (14)	Retro	40	40	60.55 ± 7.23	59.83 ± 7.7	SML+bevacizumab	Bevacizumab	11.29 ± 2.35	11.29 ± 2.35	0 and 3.6.9.12 mo:①②③④⑤
El Matri 2021 (8)	Retro	49	49	67.7 ± 5.23	61.3 ± 4.11	SML+bevacizumab	Bevacizumab	12	12	0 and 12 mo:①②③④⑤
Koushan 2022 (20)	RCT	15	15	59.8 ± 9.47	58.8 ± 9.28	SML+aflibercept	Aflibercept+sham laser	12	12	0 and 6.12 mo:①②③⑤

T indicates the experimental group (SML plus anti-VEGF injection); C indicates the control group (anti-VEGF monotherapy).

RCT, randomized controlled trial; Retro, retrospective study; SML, subthreshold micropulse laser; HD-SDM, high-density micropulse photocoagulation. Anti-vascular endothelial growth factor (Anti-VEGF) agents include Ranibizumab, Conbercept, Aflibercept, Bevacizumab.

Outcomes include ①BCVA, best corrected visual acuity; ②CMT, central macular thickness; ③Annual frequency of anti-VEGF agent injections; ④Complications; ⑤Annual SML applications.

**Table 2:** Basic characteristics of included literature (2).

Study	Interventions		HbA1c (%)		Baseline BCVA		Baseline CMT (um)	
Author(year)	anti-VEGF agent	SML(nm)	T	C	T	C	T	C
Huang KQ 2022 (11)	Ranibizumab	577	6.20 ± 1.08	5.88 ± 0.90	51.00 ± 2.61*	51.50 ± 2.76*	476.23 ± 23.02	468.42 ± 24.53
Li WQ 2019 (15)	Conbercept	577	NA	NA	57.9 ± 12.4*	59.0 ± 16.0*	427.8 ± 129.4	441.0 ± 135.7
Sun GL 2017 (12)	Ranibizumab	810	NA	NA	0. 45 ± 0. 20	0. 42 ± 0. 10	484. 92 ± 94. 43	479.12 ± 89.58
Wu Q 2021 (16)	Ranibizumab	577	NA	NA	57. 9 ± 8. 7*	57. 9 ± 9. 0*	422. 2 ± 23. 0	421. 9 ± 22. 9
Zhang Q 2021 (17)	Aflibercept	577	6.9 ± 0.8	7.0 ± 1.0	0.88 ± 0.30	0.91 ± 0.31	404.12 ± 102.31	420.90 ± 94.21
Zheng LL 2023 (18)	Conbercept	577	NA	NA	0.53 ± 0.08	0.55 ± 0.09	379.98 ± 49.56	375.36 ± 45.64
Zhou JX 2023 (13)	Conbercept	577	7.51 ± 1.32	7.47 ± 1.40	0.55 ± 0.03	0.56 ± 0.04	434.58 ± 10.88	437.36 ± 11.35
Abouhussein 2020 (7)	Aflibercept	577	8.7 ± 1.1	8.2 ± 1.2	0.76 ± 0.23	0.70 ± 0.24	469.6 ± 78.0	457.9 ± 82.2
Khattab 2019 (9)	Aflibercept	577	NA	NA	35 ± 9.9*	31. 7 ± 9.3*	457.1 ± 22.6	462 ± 31.2
Kanar 2019 (19)	Aflibercept	577	7.97 ± 2.47	8.02 ± 2.43	0.40 ± 0.09	0.38 ± 0.10	466.07 ± 71.79	451.28 ± 44.85
Altınel 2021 (14)	Bevacizumab	577	6.94 ± 0.53	6.89 ± 0.61	0.38 ± 0.21	0.39 ± 0.23	379.2 ± 70.25	384.68 ± 64.11
El Matri 2021 (8)	Bevacizumab	577	7.70 ± 0.81	7.60 ± 0.62	0.692 ± 0.35	0.598 ± 0.42	479.1 ± 14.3	359.9 ± 22.9
Koushan 2022 (20)	Aflibercept	532	NA	NA	0.36 ± 0.21	0.38 ± 0.14	457.8 ± 92.8	433.4 ± 103.5

T indicates the experimental group (SML plus anti-VEGF injection); C indicates the control group (anti-VEGF monotherapy).

SML, subthreshold micropulse laser; HbA1c, glycated hemoglobin A1c; BCVA, best corrected visual acuity; CMT, central macular thickness. NA indicates not available.

*indicates ETDRS visual acuity; unmarked indicates LogMAR visual acuity.

### 3.2 Quality evaluation

**Figure 2:**
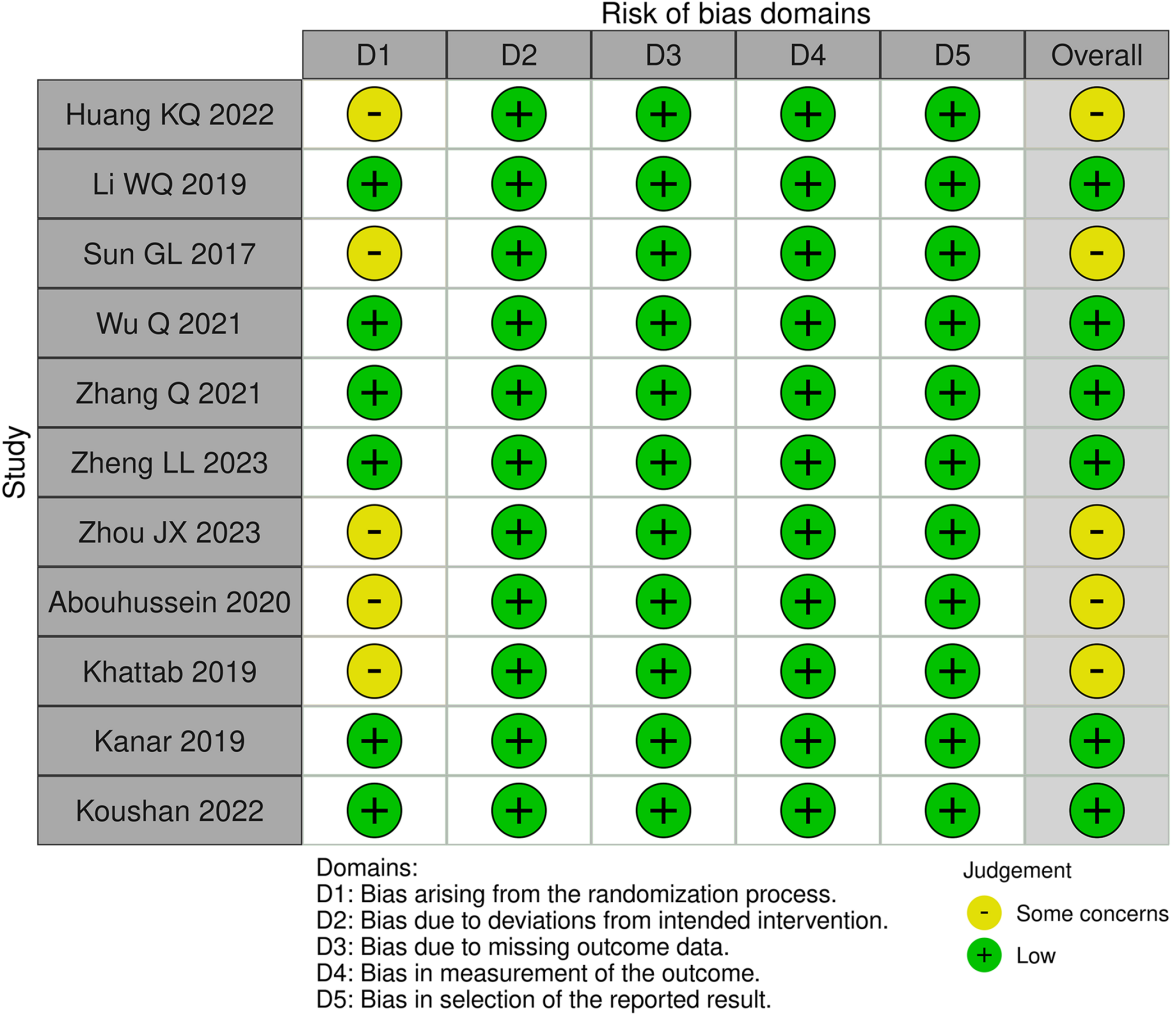
The assessment of risk of bias for RCTs (ROB 2 tool).

**Figure 3:**
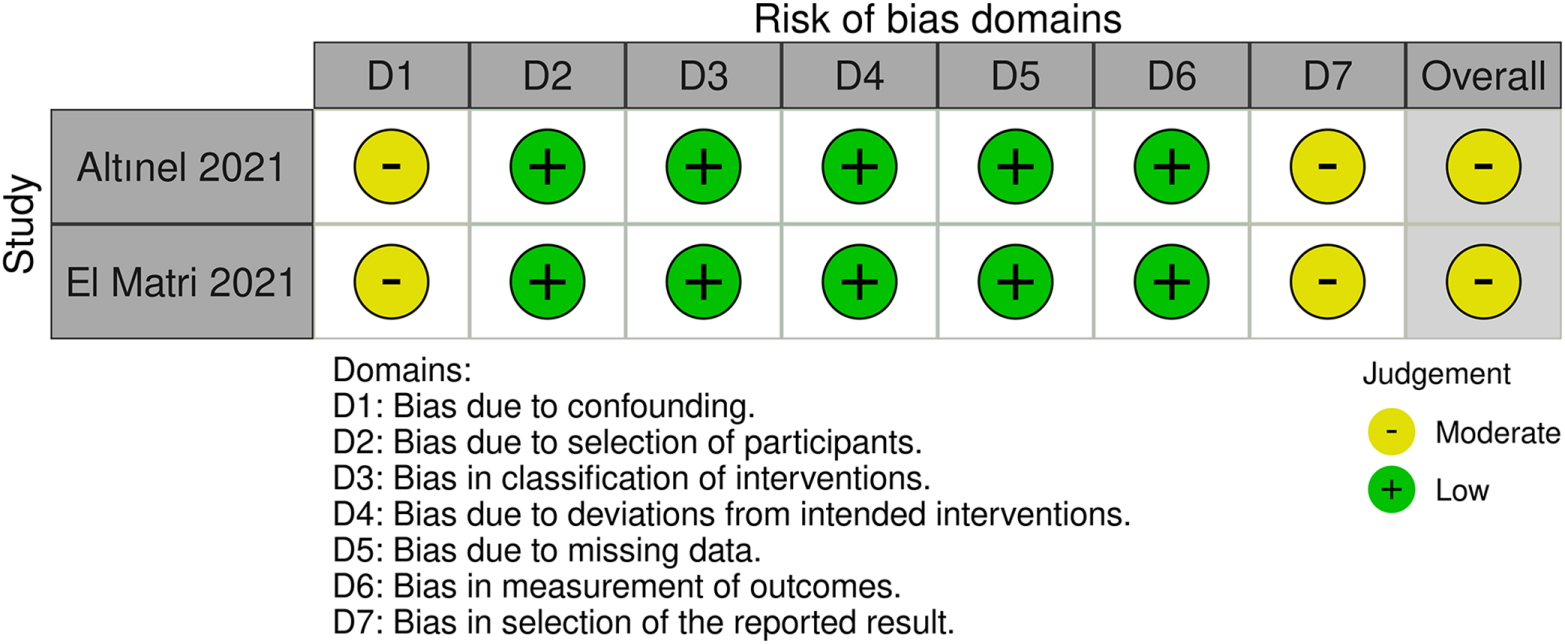
The assessment of risk of bias for retrospective studies (ROBINS I tool).

Of these 11 randomized controlled trials, RoB2 assessments yield four studies (7, 11–13) recognized as having a lack of allocation concealment during randomization, while one research (9) indicated probable discrepancies in baseline data between the two groups post-randomization. Two retrospective studies (8, 14) were assessed by the ROBINS-I tool. Both studies were classified as ‘moderate risk’, mostly due to the absence of significant confounding information in the original papers, such as systemic comorbidities and the severity of diabetic retinopathy. This may give rise to worries about the possibility of confounding bias. A summary of the risk of bias assessments for the randomized trials (**Figure 2**) and the retrospective investigations (**Figure 3**) is presented, respectively.

### 3.3 Meta-analysis

#### 3.3.1 Best corrected visual acuity (BCVA)

The BCVA of the 13 papers included in the current study comprised 4 studies for ETDRS visual acuity (9, 11, 15, 16), and 9 articles for LogMAR visual acuity (7, 8, 12–14, 17–20).

##### 3.3.1.1 ETDRS visual acuity

The heterogeneity test showed I^2^ = 0.0% (< 50%) and P = 0.733 (> 0.1) for Cochran’s Q test, indicating that there was no heterogeneity between the included literature. A fixed-effect model was selected for the meta-analysis. Overall, the results indicated that the visual acuity of the experimental group was superior to that of the control group, but with no statistically significant difference (SMD = 0.1; 0.95% Cl: -0.04, 0.24; P = 0.154 > 0.05). Subgroup analyses were conducted at various observation times, revealing that ETDRS visual acuity improved in the experimental group compared to the controls at different time points; however, the difference was not statistically significant (P = 0.669, 0.378, 0.498, and 0.338 for the 3, 6, 9, and 12 months, respectively) (**Table 3**).

**Table 3:** Meta-analysis of the BCVA (ETDRS visual acuity).

Months	Inclusion of literature (article)	Heterogeneity		Statistical Method	Effect Estimate [95%CI]	Overall effect
		I^2^	Cochran’s Q test-P			
3	4 (9, 11, 15, 16)	6.1%	0.363	Fixed	0.05 (-0.20,0.31)	P=0.669
6	4 (9, 11, 15, 16)	0.0%	0.565	Fixed	0.11 (-0.41,0.36)	P=0.378
9	3 (11, 15, 16)	0.0%	0.569	Fixed	0.10 (-0.19,0.38)	P=0.498
12	2 (9, 15)	49.4%	0.160	Fixed	0.17 (-0.18,0.53)	P=0.338
overall	–	0.0%	0.733	Fixed	0.10 (-0.04,0.24)	P=0.154

##### 3.3.1.2 LogMAR visual acuity

**Figure 4:**
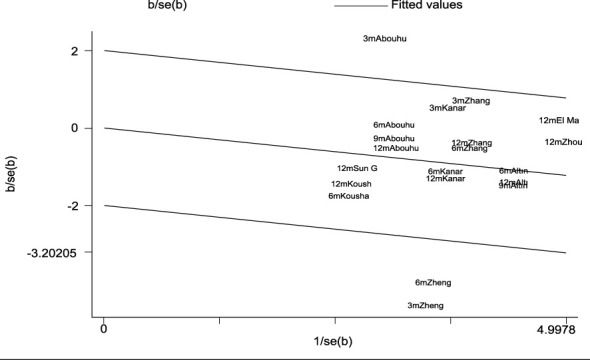
Galbraith plots for LogMAR BCVA.

The heterogeneity test indicated I^2^ = 55.0% (> 50%) and P = 0.001 (< 0.1) for Cochran’s Q test, suggesting the presence of heterogeneity among the literature selected for this study. Consequently, we performed a meta-regression analysis based on sample size (n < 30 vs. n ≥ 30), baseline central macular thickness (CMT ≥ 400 µm vs. CMT < 400 µm), and SML laser type (577 nm laser vs. others), revealing that those variables did not account for the observed heterogeneity (all P > 0.05). Furthermore, a Galbraith star plot was created (**Figure 4**). This figure illustrates that the heterogeneity of two studies, Zheng LL (2023) and Abouhussein (2020), is significant. Following the exclusion of these publications, the results of the meta-analysis covering a total of seven studies (8, 12–14, 17, 19, 20) revealed I^2^ = 0.00% (< 50%) and P = 0.784 (> 0.1) for Cochran’s Q test; effect sizes were subsequently combined using a fixed-effect model. Overall, our findings demonstrated that LogMAR visual acuity in the experimental group was superior to that in the controls, with a statistically significant difference (SMD = -0.19; 95% CI: -0.32, -0.07; P = 0.003 < 0.05). Subgroup analysis conducted at different time intervals showed that the experimental group achieved improved LogMAR visual acuity compared to the control group at both 6 and 12 months, with statistically significant differences (P = 0.026 and 0.041 for 6 and 12 months, respectively) (**Table 4**).

**Table 4:** Meta-analysis of the BCVA (LogMAR visual acuity).

Months	Inclusion of literature (article)	Heterogeneity		Statistical Method	Effect Estimate [95%CI]	Overall effect
		I^2^	Cochran’s Q test-P			
3	3 (14, 17, 19)	0.0%	0.371	Fixed	-0.02 (-0.29,0.26)	P=0.906
6	4 (14, 17, 19, 20)	0.0%	0.707	Fixed	-0.29 (-0.56,-0.03)	P=0.026^*^
9	1 (14)	NA	NA	Fixed	-0.36 (-0.08,0.09)	P=0.114
12	7 (8, 12–14, 17, 19, 20)	0.0%	0.722	Fixed	-0.19 (-0.38,-0.01)	P=0.041^*^
overall	–	0.0%	0.784	Fixed	-0.19 (-0.32,-0.07)	P=0.003^*^

NA indicates heterogeneity test not applicable due to inclusion of only one publication.

*indicates a statistical difference of P < 0.05.

#### 3.3.2 Central macular thickness

The heterogeneity test displayed I^2^ = 42.1% (< 50%) but P = 0.0061 (< 0.1) for Cochran’s Q test, indicating significant heterogeneity among the included studies. We then conducted a meta-regression analysis, accounting for the sample size, baseline CMT, and the laser type of SML. The results demonstrated that neither sample size nor SML laser type (both P > 0.05) contributed to the observed heterogeneity. However, baseline CMT emerged as a significant factor (P = 0.000 < 0.05) influencing this variability.

**Figure 5:**
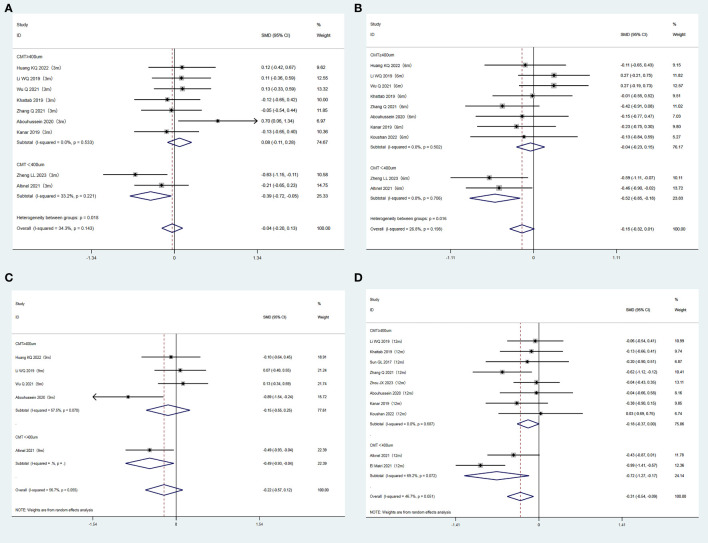
**(A)** Forest plots comparing subgroups (CMT ≥ 400 um/CMT < 400 um) for CMT at 3 months after treatment in the experimental and control groups (Fixed-effect model). **(B)** Forest plots comparing subgroups (CMT ≥ 400 um/CMT < 400 um) for CMT at 6 months after treatment in the experimental and control groups (Fixed-effect model). **(C)** Forest plots comparing subgroups (CMT ≥ 400 um/CMT < 400 um) for CMT at 9 months after treatment in the experimental and control groups (Random-effect model). **(D)** Forest plots comparing subgroups (CMT ≥ 400 um/CMT < 400 um) for CMT at 12 months after treatment in the experimental and control groups (Random-effect model).

Participants were classified into two subgroups based on the baseline CMT (CMT ≥ 400 µm and CMT < 400 µm). In the subgroup with baseline CMT < 400µm, the CMT value was consistently and significantly reduced in the experimental group compared to the control group at each observation time point (P = 0.024, 0.003, 0.031, and 0.011 for the 3, 6, 9, and 12 months, respectively). In contrast, no significant differences in the CMT levels were observed between the two groups at any time point for the subgroup with baseline CMT ≥ 400 µm (P = 0.406, 0.673, 0.468, and 0.056 for the 3, 6, 9, and 12 months, respectively). The results are summarized in **Figures 5A–D**.

#### 3.3.3 The annual frequency of anti-VEGF drugs injections

**Figure 6:**
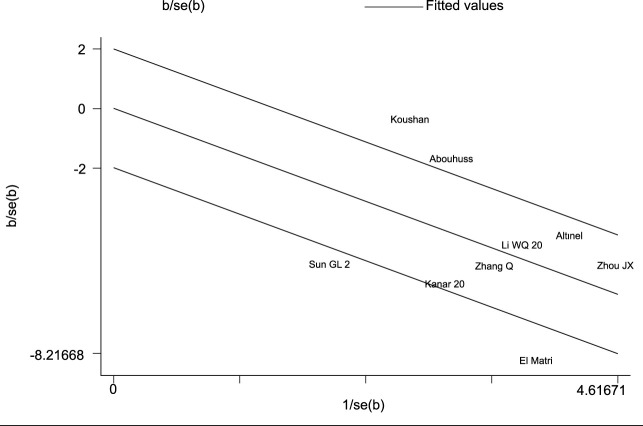
Galbraith plots for the number of annual anti-VEGF drugs injections.

**Figure 7:**
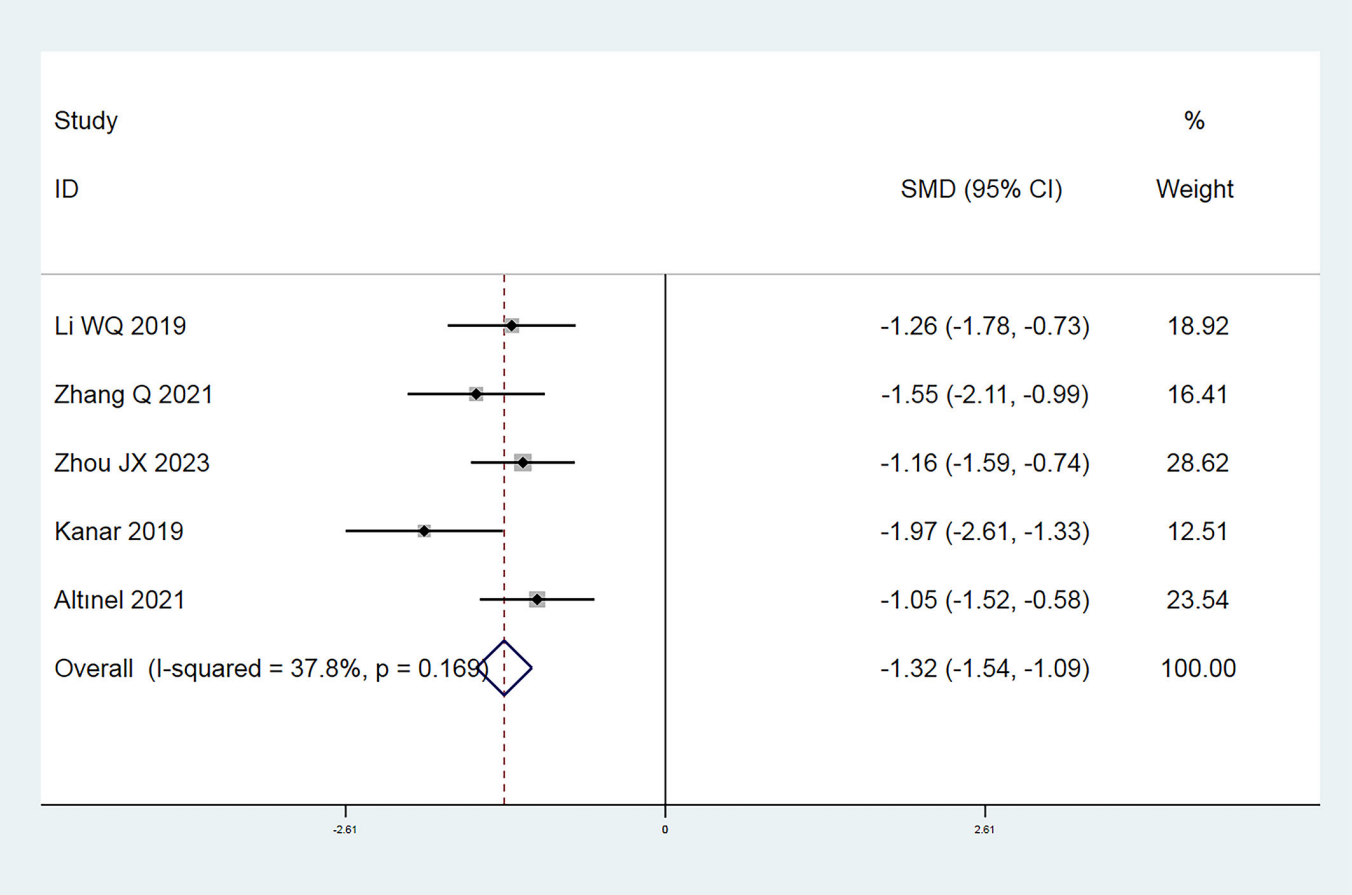
Forest plot comparing the number of annual anti-VEGF drugs injections in the experimental and control groups (Fixed-effect model).

In this investigation, a total of 9 publications (7, 8, 12–15, 17, 19, 20) reported the annual frequency of vitreous anti-VEGF drugs injections. Following the heterogeneity test, I² was judged to be 80.3%, showing high heterogeneity among the included studies (P = 0.000 < 0.1 from Cochran’s Q test). We took a meta-regression analysis considering sample size, CMT, and SML laser types and indicated that none of these parameters significantly contributed to the heterogeneity (all P > 0.05). A Galbraith star diagram was later constructed, and the findings are given in **Figure 6**. Particularly, investigations by Sun GL (2017), Abouhussein (2020), El Matri (2021), and Koushan (2022) demonstrated high heterogeneity. After eliminating these articles, a meta-analysis was undertaken on 5 remaining studies (13–15, 17, 19). The heterogeneity test gave I² = 37.8% (< 50%) with P = 0.169 (> 0.1), thus we proceeded with a combined effect size estimate using a fixed-effect model. Overall, the frequency of annual vitreous anti-VEGF drugs injections in the experimental group was considerably lower than that in the control group (SMD = -1.32; 95% Cl: -1.54, -1.09; P = 0.000 < 0.05) (**Figure 7**).

#### 3.3.4 The annual frequency of SML applications

Six research papers (8, 13, 14, 17, 19, 20) tracked the annual frequency of SML applications within the combined group. The experimental designs vary across investigations (refer to **Supplementary Data Sheet 2** for detailed methodologies). Most studies adopted a threshold-based strategy in which the experimental group was monitored monthly following the initial SML treatment combined with vitreous injection of anti-VEGF agents (once a month for a total of 3 doses as a loading dose). SML should be re-administered if the loss of vision and/or the change of CMT rose above the defined threshold value, but this intervention could not occur earlier than 2 or 3 months after the prior SML treatment. The results showed that an average annual SML application frequency ranged from 1.41 ± 0.37 to 3.4 ± 1.4 times (range: 1-4 times).

#### 3.3.5 Complications

A total of seven articles (7–9, 12, 14, 17, 18) reported complications in our research. Three papers (12, 14, 17) observed no ocular or systemic complications during the follow-up. Two papers (7, 8) reported that there were no injection-related complications apart from mild subconjunctival hemorrhage at the injection site. No significant SML laser scars were observed in the combined treatment group. One study (9) described a mild inflammatory reaction in the eye postoperatively, which could subside within a week with topical steroid drops. Only one paper (18) provided data on the incidence of complications, which were eye pain, dry eye, elevated intraocular pressure, and foreign body sensation. However, the total incidence was not statistically different between the two groups (X^2^ = 0.185, P = 0.667).

### 3.4 Sensitivity analysis

Sensitivity evaluations were conducted for three outcome indicators including BCVA (ETDRS visual acuity and LogMAR visual acuity), CMT, and the frequency of annual vitreous anti-VEGF drugs injections using the one-by-one exclusion method. The results showed that the exclusion of any one piece of literature would not cause significant interference to this meta-analysis, indicating that our study has good stability (**Supplementary Data Sheet 3**).

### 3.5 Publication bias test

A Begg’s test was conducted with Stata SE15 software. The results revealed that there was no publication bias for the outcome measures of BCVA (ETDRS visual acuity and LogMAR visual acuity), CMT, and the annual frequency of anti-VEGF drugs injections (**Table 5**).

**Table 5:** Publication bias test of included articles.

Research content	Inclusion of literature (article)	The P value of Begg’s Test
BCVA (ETDRS)		
3m	4	0.734
6m	4	0.734
9m	3	0.296
12m	2	1.000
BCVA (LogMAR)		
3m	3	1.000
6m	4	0.308
9m	1	NA
12m	7	0.133
CMT		
3m	9	0.602
6m	10	1.000
9m	5	0.462
12m	10	0.474
**Numbers of annual vitreous injection**	5	0.086

BCVA, best corrected visual acuity; CMT, central macular thickness.

NA indicates that due to the inclusion of only one publication, the publication bias test is not applicable.

## 4 Discussion

Diabetic macular edema (DME) is the primary cause of visual impairment for diabetic patients. Vitreous injection of anti-VEGF drugs is currently the first-line treatment to control DME. However, this drug has a short half-life, requires multiple injections to maintain efficacy, and is expensive, thereby increasing the financial burden on patients. In addition, recurrent vitreous injections may carry certain risks, including cardiovascular and cerebrovascular accidents, as well as ocular complications such as glaucoma, cataracts, retinal detachment, endophthalmitis, and others (21). Therefore, reducing the frequency of injection and minimizing the complications on the basis of ensuring the efficacy is an urgent problem to be solved at present. A number of scholars (22–24) have advised the use of anti-VEGF drugs intravitreal injection combined with retinal laser photocoagulation as a treatment for DME. This strategy has been demonstrated to considerably reduce macular edema and improve postoperative visual acuity, while also reducing the amount of vitreous injections necessary (25). It is important to note that traditional photocoagulation treatment still involves a destructive process on the retina. This process can result in a number of adverse effects, including permanent loss of photoreceptors, choroidal neovascularization, and subretinal fibrosis (26). Different from the traditional retinal laser photocoagulation, in which the laser action time is equal to the whole exposure time, the subthreshold micropulse laser (SML) consists of a series of complete and repeatable high-frequency pulses, and due to the small duty cycle and long intervals between the pulses, the laser energy cannot be accumulated continuously, so the damage to the retina caused by subthreshold micropulse lasers is limited. What’s more, the heat effect it created is only limited to the retinal pigment epithelial (RPE) layer with no visible retinal laser spot (6), thus reducing the burn injury on the retinal neurosensory layer and deep choroidal capillaries (12). In addition, the SML can produce biomodulatory effects on the RPE cells. The SML promotes the expression of heat shock protein 70 (HSP70) in the RPE cells (27), which in turn folds the damaged RPE cells, prevents apoptosis, and blocks inflammatory pathways. This contributes to the remodeling of the RPE cells and accelerates the absorption of macular edema. Furthermore, micropulse laser treatment has been demonstrated to reduce the expression of various growth factors, including vascular endothelial growth factor (VEGF), transforming growth factor-β (TGF-β), pigment epithelium-derived factor (PEDF), and basic fibroblast growth factor (bFGF). As well as, the SML contributes to the upregulation of angiogenesis inhibitors, which in turn inhibit retinal neovascularization (28).

Our research indicates that in the LogMAR visual acuity group, postoperative visual acuity improved in the combination therapy group compared to the monotherapy group at 6 and 12 months and the annual frequency of vitreous injections was significantly decreased in the combination group. As anti-VEGF drugs have the capacity to rapidly reduce macular edema, the implementation of micropulse laser treatment in most trials occurred within 1-2 weeks after vitreous injection of anti-VEGF drugs. At the moment, the reduction of central macular thickness avoids the formation of a barrier owing to fluid accumulation in the macula, which was more favorable to the micropulse laser’s direct action on the RPE cells (15). In addition, vitreous injection of anti-VEGF drugs can reduce vascularization, leakage, edema, and inflammation after photocoagulation, minimizing retinal damage from the SML laser (23, 29). Attention is drawn to the fact that anti-VEGF drugs have the disadvantage of a short duration of action, thus requiring repeated injections to maintain efficacy, whereas micropulse lasers may take several months to show therapeutic effects (30), thereby combining the two methods to achieve a complementary advantage. What’s more, the cost of one SML session is roughly $60, but the cost of one vitreous anti-VEGF drug injection is approximately $430, and the combination of them considerably decreases the financial burden of patients.

Unfortunately, in the ETDRS visual acuity group, there was no statistically significant difference for BCVA between the two groups. The ETDRS visual acuity chart is a standardized version based on the design of the LogMAR principles, with each line of letters corresponding to a change in visual acuity of 0.1 log units. ETDRS visual acuity measurement is standardized by controlling the test distance (31, 32), density of letter arrangement (33), standardization of illumination, etc. Compared with conventional LogMAR visual acuity, ETDRS visual acuity can control the measurement error within ± 0.02 logMAR (two lines of letters) (34) and is more sensitive. What’s more, although the difference was not statistically significant, we also observed an improvement of ETDRS visual acuity in the combination therapy group compared to the monotherapy group at all observation time points.

It is documented that the impact of micropulse laser is closely related to central macular thickness (CMT) (11, 15), and the efficacy of micropulse laser monotherapy in eyes with CMT > 400 μm is inferior. Therefore, combining micropulse laser with anti-VEGF drugs injections is required (35), which is consistent with the findings of our study. Based on clinical observation, when macular oedema is more severe, the distribution of micropulsed laser energy is more diffused in the targeted tissues, which makes it difficult to penetrate the edema to operate on the RPE cells, and thus the efficacy of the treatment is not satisfactory (13).

At present, primarily two kinds of lasers are employed for clinical treatment: the 577 nm yellow laser and the 810 nm near-infrared laser. Both lasers are suitable for diabetic macular edema. The 810-nm SML has an effect on melanocytes in both the RPE layer and choroid. In contrast, the 577-nm SML has a more targeted effect, especially affecting the melanocytes in the RPE layer. The 577-nm laser has the advantage of scattering slightly less than the 810-nm laser, allowing for more focused energy in the RPE layer, which lessens the laser strength and shortens pulse duration (36). Furthermore, since the absorption spectrum of lutein does not contain 577 nm yellow light, 577 nm SML can be employed in close proximity to the central concave of the macula, making 577 nm laser the favored option (37). Although there is a bit of research on the 810 nm near-infrared laser, recent findings indicate that at a duty cycle of 5%, the two lasers have comparable therapeutic efficacy (36, 38). The results of our study reveal that the type of laser employed does not alter the change of central macular thickness. However, the lack of non-577 nm laser literature may introduce a publication bias. Therefore, further investigation is required to confirm whether laser type could affect therapeutic efficacy.

The adverse effects of combination therapy appear to be more mild in the current research, with the most common postoperative complications including mild subconjunctival hemorrhage, a light ocular inflammatory reaction, and/or ocular surface discomfort.

## 5 Limitations

Some important limitations should be considered. First, the baseline data revealed a discrepancy in age between the experimental and control groups. Although age is a relevant factor in the development of DME (39, 40), it has been demonstrated in the study by Zhu TT et al. (41) that age is not an independent risk factor for the prognosis of DME treated with conbercept by a multifactorial logistic regression analysis. Ayumi et al. (42) investigated the factors associated with the efficacy of aflibercept or ranibizumab in treating DME. They also failed to find a statistically significant difference between the age of the good response group (> 20% decrease in CMT) and the poor response group (≤ 20% decrease in CMT) (P = 0.061). In addition, the study by Alshalan et al. (43) showed that although there were differences between the mean improvement in BCVA and the mean change in the central subfield thickness (CST) decreased with DME patients of different ages (age ≤ 60 years vs. age > 60 years) treated with anti-VEGF agents, the differences were not statistically significant (P = 0.5429, 0.08, respectively). Therefore, we believe that the difference in baseline age may not have a major impact on outcome measures such as CMT and visual acuity between the two groups. Second, due to missing or incomplete information in the literature, the clinical heterogeneity sources were not detected for certain outcome indicators. Factors such as the severity of diabetic retinopathy (44), lens status (45), and other factors may contribute to the clinical heterogeneity. In the future, we need to include as much literature as possible and find out the sources of clinical heterogeneity.

## 6 Conclusion

In conclusion, we suggest that subthreshold micropulse laser (SML) combined with vitreous anti-VEGF drugs injections can significantly reduce macular edema and improve visual acuity in patients of DME, especially in those with CMT < 400 um. In addition, combined therapy can significantly reduce the frequency of anti-VEGF drugs injections with fewer postoperative complications, and SML can be repeated, which significantly reduces the economic burden on patients.
