# Supplementary material for: Evaluating the efficacy of subthreshold micropulse laser combined with anti-VEGF drugs in the treatment of diabetic macular edema: a systematic review and meta-analysis
Source: Front Endocrinol (Lausanne). 2025 Mar 28;16:1553311. doi: 10.3389/fendo.2025.1553311 (PMC11985442; doi:10.3389/fendo.2025.1553311)
Supplement: Supplementary file 1 [file DataSheet1.docx]

***Supplementary Material***

**Search strategy**

- **English publications**

search terms through PubMed (as a example)：

Search: (((((((((((((((Macular Edema[Title/Abstract]) OR (Edema, Macular[Title/Abstract])) OR (Irvine-Gass Syndrome[Title/Abstract])) OR (Irvine Gass Syndrome[Title/Abstract])) OR (Syndrome, Irvine-Gass[Title/Abstract])) OR (Cystoid Macular Edema, Postoperative[Title/Abstract])) OR (Macular Edema, Cystoid[Title/Abstract])) OR (Edema, Cystoid Macular[Title/Abstract])) OR (Cystoid Macular Dystrophy[Title/Abstract])) OR (Macular Dystrophy, Dominant Cystoid[Title/Abstract])) OR (Central Retinal Edema, Cystoid[Title/Abstract])) OR (Cystoid Macular Edema[Title/Abstract])) AND (Diabetic[Title/Abstract])) AND ((((((((((((((((((((((((((Ranibizumab[Title/Abstract]) OR (RhuFab V2[Title/Abstract])) OR (V2, RhuFab[Title/Abstract])) OR (Lucentis[Title/Abstract])) OR (Bevacizumab[Title/Abstract])) OR (Mvasi[Title/Abstract])) OR (Bevacizumab-awwb[Title/Abstract])) OR (Bevacizumab awwb[Title/Abstract])) OR (Avastin[Title/Abstract])) OR (conbercept[Title/Abstract])) OR (aflibercept[Title/Abstract])) OR (VEGF Trap - regeneron[Title/Abstract])) OR (VEGF Trap-Eye[Title/Abstract])) OR (VEGF-Trap[Title/Abstract])) OR (eylea[Title/Abstract])) OR (Zaltrap[Title/Abstract])) OR (AVE 0005[Title/Abstract])) OR (AVE0005[Title/Abstract])) OR (AVE-0005[Title/Abstract])) OR (AVE 005[Title/Abstract])) OR (AVE005[Title/Abstract])) OR (AVE-005[Title/Abstract])) OR (ZIV-aflibercept[Title/Abstract])) OR (anti-VEGF[Title/Abstract])) OR (anti-vascular endothelial growth factor[Title/Abstract])) OR (anti-VEGF therapy[Title/Abstract]))) AND (((((((((((micropulse laser[Title/Abstract]) OR (subthreshold micropulse laser[Title/Abstract])) OR (subthreshold micropulse laser photocoagulation[Title/Abstract])) OR (subthreshold laser[Title/Abstract])) OR (High-density micropulse photocoagulation[Title/Abstract])) OR (subthreshold diode micropulse laser[Title/Abstract])) OR (subthreshold grid laser[Title/Abstract])) OR (micropulsed yellow laser[Title/Abstract])) OR (laser photocoagulation[Title/Abstract])) OR (laser coagulation[Title/Abstract])) OR (laser thermocoagulation[Title/Abstract]))) AND (((randomized controlled trial[Publication Type]) OR (randomized[Title/Abstract])) OR (placebo[Title/Abstract]))

- **Chinese publications**

search terms through China Biomedical Literature Database (CBM) (as a example)：

| **No.** | **Search strategy** | **Outcome** |
| --- | --- | --- |
| 5 | (#4) AND (#3) AND (#2) AND (#1) | 152 |
| 4 | “Randomized controlled” [common field: smart] OR “Randomized controlled trial” [common field: smart] OR “Randomized” [common field: smart] OR “RCT” [common field: smart] | 1919182 |
| 3 | “Micropulse laser” [common field:smart] OR “Subthreshold micropulse diode laser” [common field:smart] OR “Subthreshold micropulse laser” [common field:smart] OR “High-density micropulse laser” [common field:smart] OR “Laser photocoagulation” [common field:smart] | 2697 |
| 2 | “Anti-VEGF” [Common Fields: smart] OR “Anti-vascular endothelial growth factor” [Common Fields: smart] OR “Anti-VEGF therapy” [Common Fields: smart] OR “Ranibizumab” [Common Fields: smart] OR “Bevacizumab” [Common Fields: smart] OR “Conbercept” [Common Fields: smart] OR “Aflibercept” [Common Fields: smart] | 6677 |
| 1 | “Diabetic macular edema” [common field:smart] OR “DME” [common field:smart] OR “Diabetic retinopathy” [common field:smart] OR “Macular edema” [common field:smart] OR “DM” [common field:smart] | 63267 |
