# Supplementary material for: Evaluating the efficacy of subthreshold micropulse laser combined with anti-VEGF drugs in the treatment of diabetic macular edema: a systematic review and meta-analysis
Source: Front Endocrinol (Lausanne). 2025 Mar 28;16:1553311. doi: 10.3389/fendo.2025.1553311 (PMC11985442; doi:10.3389/fendo.2025.1553311)
Supplement: Supplementary file 2 [file DataSheet2.docx]

**Detailed methodologiests for inclusion trials.**

| **Study** | **Interventions** | | **Dosage and timing** | |
| --- | --- | --- | --- | --- |
| **Author(year)** | **anti-VEGF agent** | **SML（nm）** | **only IVI** | **IVI+SML** |
| Huang KQ 2022 | Ranibizumab | 577 | 3 × monthly IVI of 0.5 mg as a loading dose, + PRN IVI | 3 × monthly IVI of 0.5 mg as a loading dose + SML 2wk after the first IVI, PRN IVI |
| Li WQ 2019 | Conbercept | 577 | 3 × monthly IVI of 0.5 mg/ 0.05mL as a loading dose, + PRN IVI | 3 × monthly IVI of 0.5 mg/ 0.05mL as a loading dose + SML within 2 weeks after the IVI ( the time from the last SML was more than 3 months ), PRN IVI |
| Sun GL 2017 | Ranibizumab | 810 | 3 × monthly IVI of 0.5 mg , + PRN IVI | 3 × monthly IVI of 0.5 mg+ HD-SDM 2 weeks after the first IVI, PRN IVI |
| Wu Q 2021 | Ranibizumab | 577 | 1 × monthly IVI of 0.5 mg , + PRN IVI | 1 × monthly IVI of 0.5 mg+ SML within 2 weeks after the IVI, PRN IVI |
| Zhang Q 2021 | Aflibercept | 577 | 3 × monthly IVI of 2 mg/0.05 mL as a loading dose, + PRN IVI ( When CMT ≤ 250um, the IVI is suspended, otherwise, the treatment is re-injected. ) | 3 × monthly IVI of 2 mg/0.05 mL as a loading dose + SML within 2 week after the 3rd injection, PRN IVI or SML ( Once the CMT level is higher than the standard line, if the last SML is less than one month, the IVI will be performed. If the last SML is more than 1 month, the SML alone will be performed. ) |
| Zheng LL 2023 | Conbercept | 577 | 3 × monthly IVI of 0.5 mg/ 0.05mL | 3 × monthly IVI of 0.5 mg/ 0.05mL + SML at 1 week after the third IVI |
| Zhou JX 2023 | Conbercept | 577 | 3 × monthly IVI of 0.5 mg/0.05 mL as a loading dose, + PRN IVI | 3 × monthly IVI of 0.5 mg/0.05 mL as a loading dose + SML within 2wk after the IVI ( the time from the last SMLP was ≥3 months ), PRN IVI |
| Abouhussein 2020 | Aflibercept | 577 | 3 × monthly IVI of 2 mg/0.05 mL as a loading dose, + PRN IVI | 3 × monthly IVI of 2 mg/0.05 mL as a loading dose + SML followed 1 month later, monthly PRN IVI |
| Khattab 2019 | Aflibercept | 577 | 3 × monthly IVI of 2 mg/0.05 mL as a loading dose, + PRN IVI ( Injections were suspended when the CMT reached 250 μm or less at any point of the follow-up. ) | 3 × monthly IVI of 2 mg/0.05 mL as a loading dose + SML within 1 week after the 3rd injection, PRN IVI ( Whenever the CMT was still above the cutoff level, injections were given alone if the last MPL session was done in <1 month. The IVI + MPL were given together (1 week apart), if the last MPL session was carried at ≥ 2 months. ) |
| Kanar 2019 | Aflibercept | 577 | 3 × monthly IVI of 2 mg/0.05 mL as a loading dose, + PRN IVI | 3×monthly IVI 2 mg/0.05 mL + SML at 1 month if CMT decreased<450 um，if>450 um a second course of one or more IVI+SML applied at 4 weeks |
| Altınel 2021 | Bevacizumab | 577 | 3×monthly IVI 1.25 mg/0.05 mL IVI + IVI PRN | 3×monthly IVI 1.25 mg/0.05 mL IVI+SML 4 weeks after the loading dose if CMT<400 um, Otherwise,if CMT was ≥400 μm, one or more IVBs wereapplied monthly until the CMT had decreased to <400 μm |
| El Matri 2021 | Bevacizumab | 577 | 3×monthly IVI 1.25 mg/ 0.05 mL + IVI PRN 4 weekly | 3×monthly IVI 1.25 mg/ 0.05 mL + SML within 1 week after thethird injection，monthly PRN IVI |
| Koushan 2022 | Aflibercept | 532 | 1×IVI 2.0 mg/0.05 mL+sham laser on the same day + IVI PRN ( At intervals no shorter than 12 weeks, study eyes + sham laser. ) | 1× IVI of 2.0 mg/0.05 mL IVI + SML on the same day + IVI PRN ( At intervals no shorter than 12 weeks, study eyes + SML ) |

Anti-vascular endothelial growth factor (Anti-VEGF) agents include Ranibizumab, Conbercept, Aflibercept, and Bevacizumab. SML, subthreshold micropulse laser; IVI, intravitreal injection of Anti-VEGF agents; IVI+SML, intravitreal injection of Anti-VEGF agent combined with subthreshold micropulse laser therapy; PRN, Pro re nata.
