# Supplementary figures and images for: Evaluating the efficacy of subthreshold micropulse laser combined with anti-VEGF drugs in the treatment of diabetic macular edema: a systematic review and meta-analysis
Source: Front Endocrinol (Lausanne). 2025 Mar 28;16:1553311. doi: 10.3389/fendo.2025.1553311 (PMC11985442; doi:10.3389/fendo.2025.1553311)

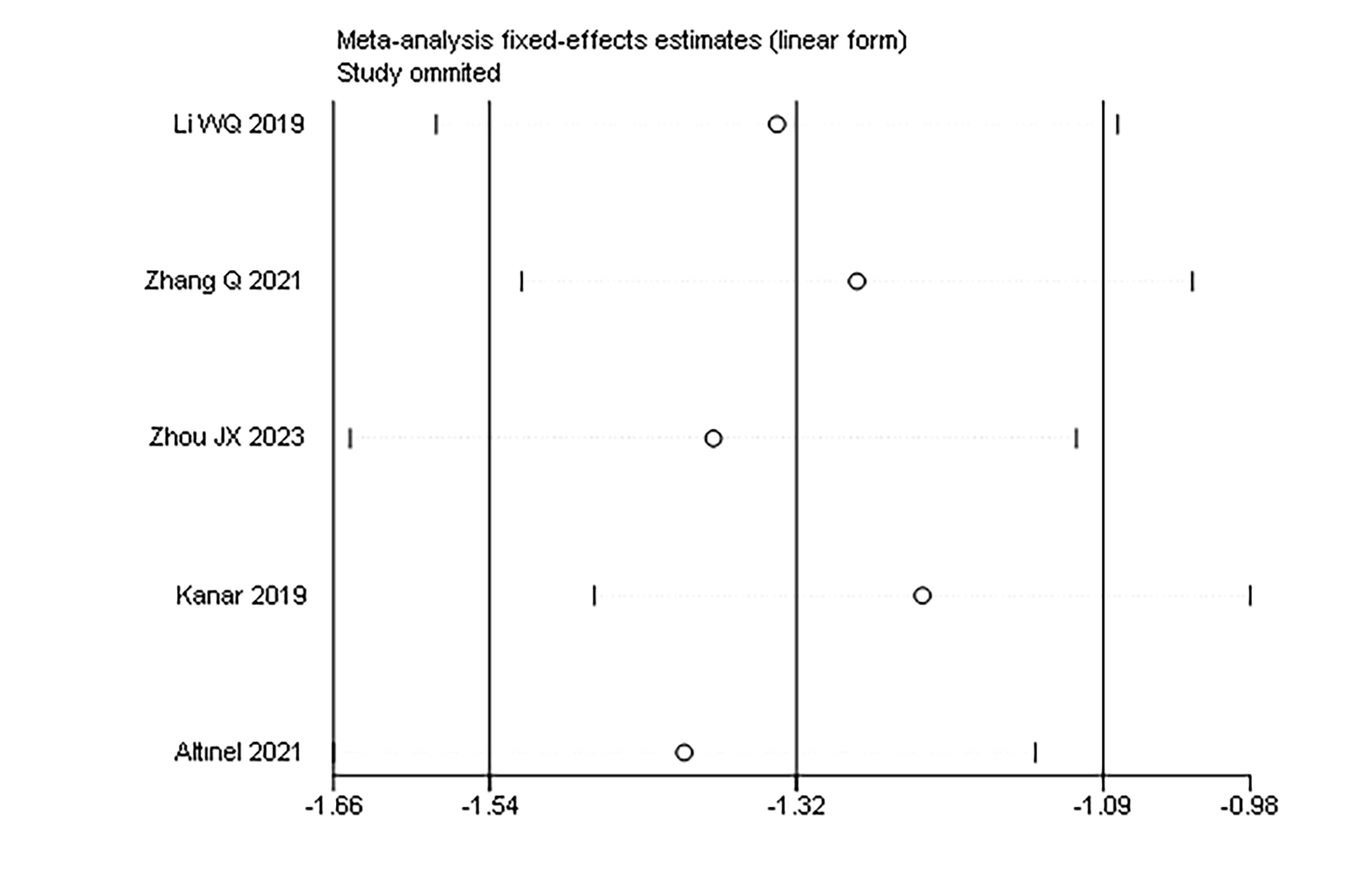

Supplement: Supplementary file 3 [file DataSheet3.zip › Original image of Sensitivity analysis/Sensitivity analysis of annual frequency of anti-VEGF drugs injections.tif]

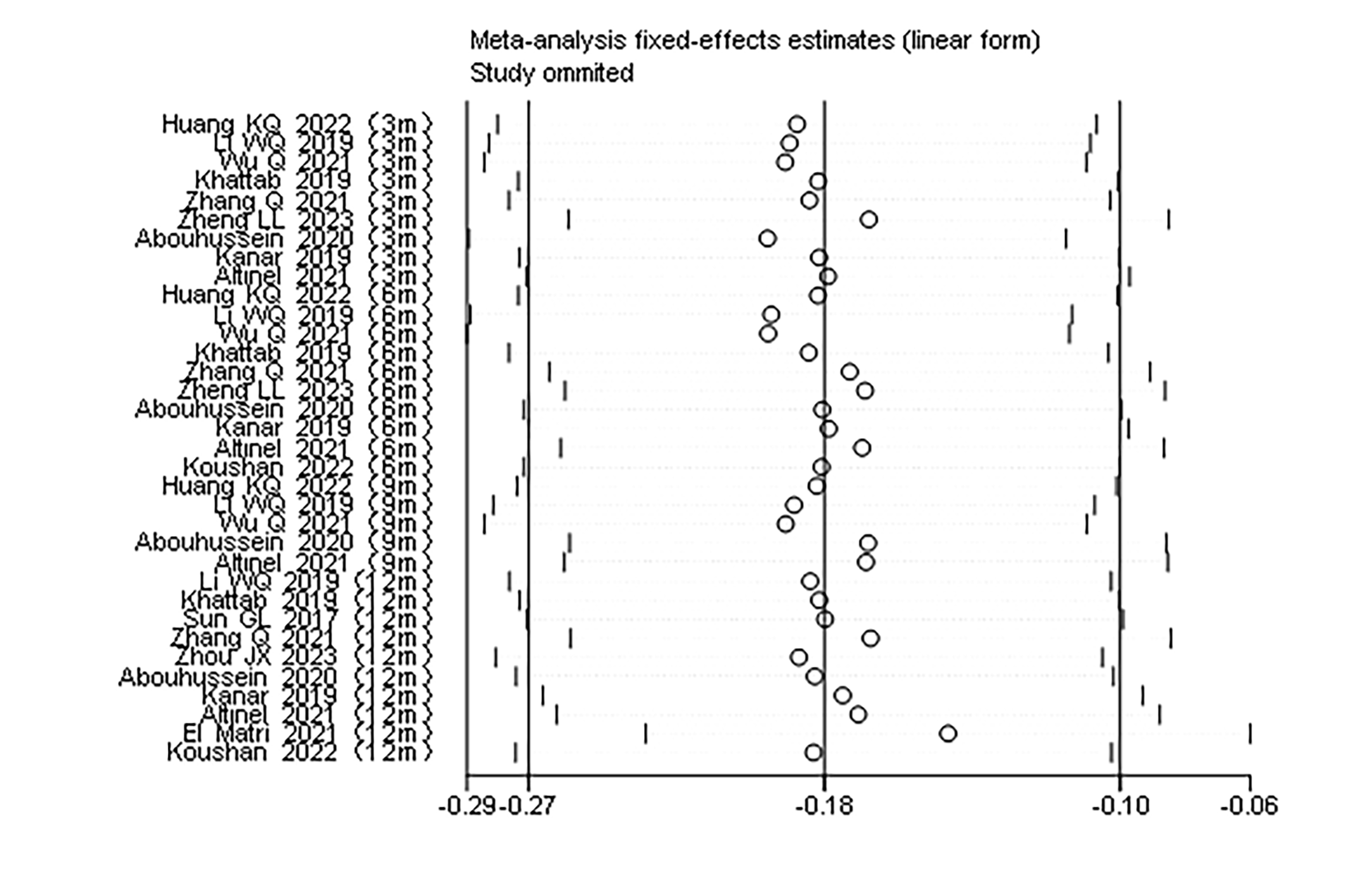

Supplement: Supplementary file 3 [file DataSheet3.zip › Original image of Sensitivity analysis/Sensitivity analysis of CMT.tif]

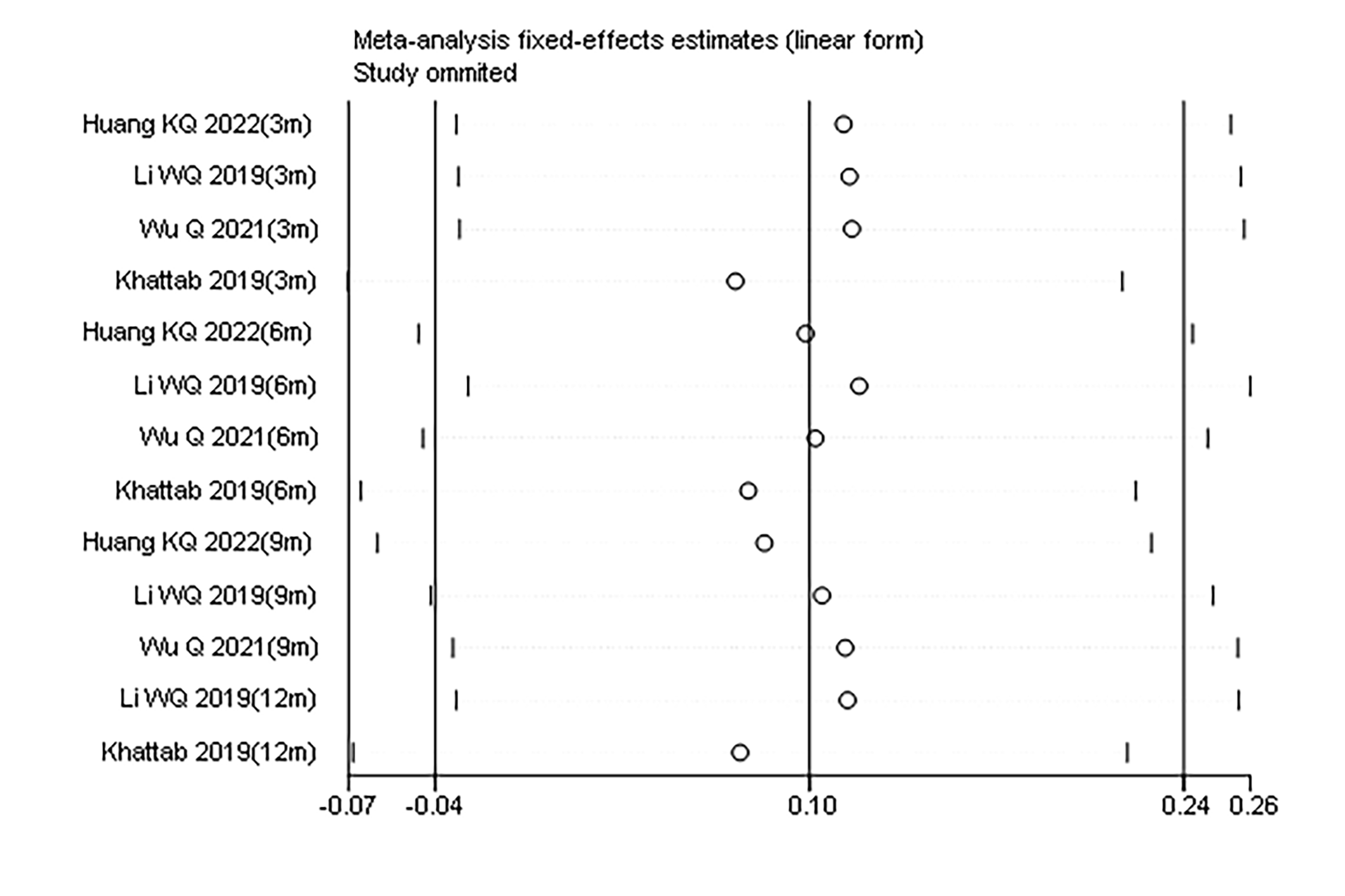

Supplement: Supplementary file 3 [file DataSheet3.zip › Original image of Sensitivity analysis/Sensitivity analysis of ETDRS visual acuity.tif]

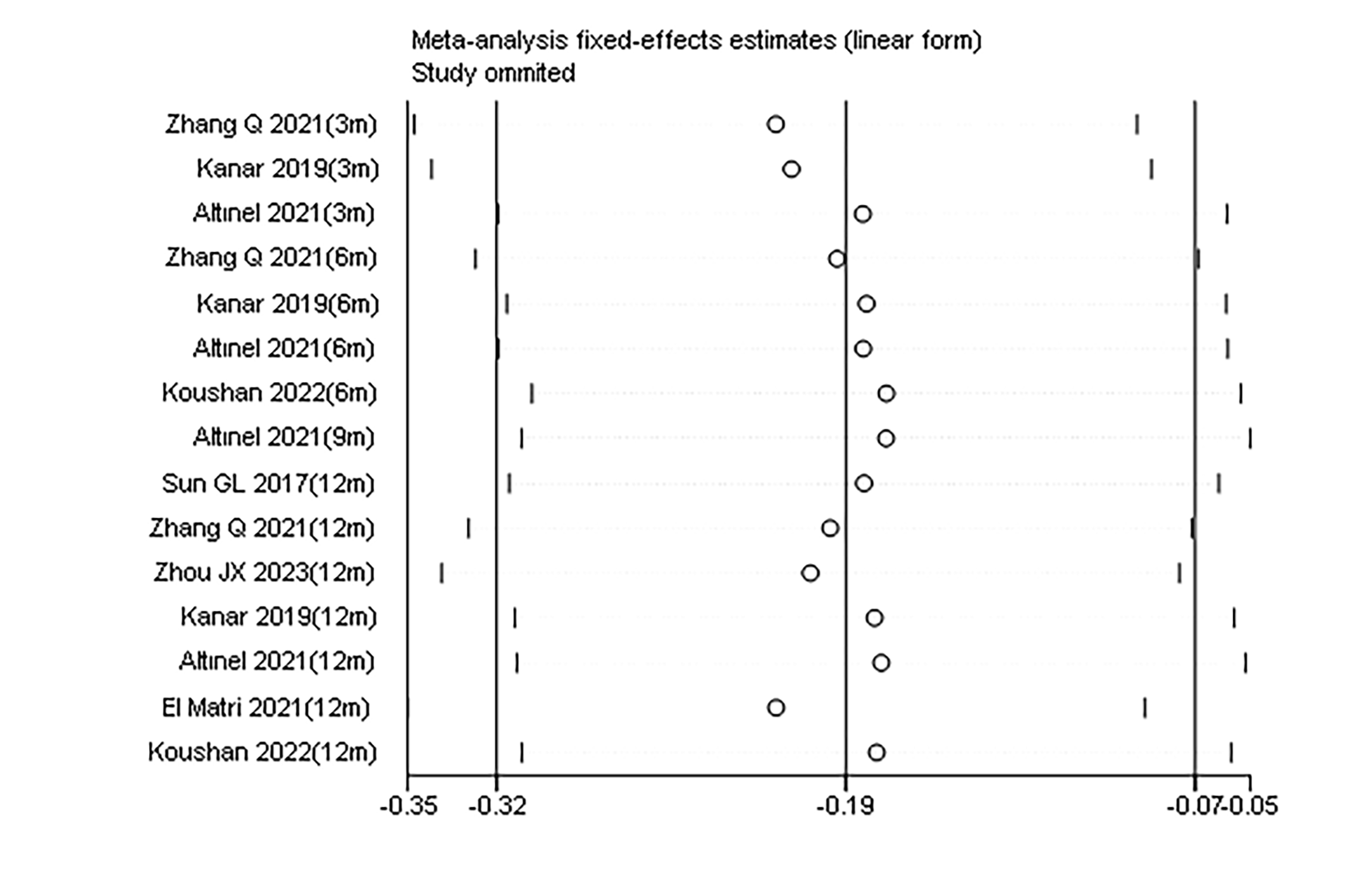

Supplement: Supplementary file 3 [file DataSheet3.zip › Original image of Sensitivity analysis/Sensitivity analysis of LogMAR visual acuity.tif]
